# Non-Destructive Intelligent Microwave Sensing of Water Content in Sand–Water Mixtures Using S-Parameter Features and PLS Regression

**DOI:** 10.3390/s26154766

**Published:** 2026-07-27

**Authors:** Mehmet Çakır

**Affiliations:** Department of Electrical-Electronics Engineering, Bayburt University, Bayburt 69000, Türkiye; mcakir@bayburt.edu.tr

**Keywords:** microwave sensing, S-parameters, water content estimation, sand–water mixtures, partial least squares regression

## Abstract

The accurate and rapid estimation of water content in granular materials is important for geotechnical engineering, construction-material evaluation, agricultural monitoring, and non-destructive material assessment. This study presents a controlled laboratory feasibility framework for estimating water content in sand–water mixtures using microwave S-parameter measurements and physically interpretable features. Sixty measurements were acquired from six nominal mixture levels containing 0%, 5%, 10%, 15%, 20%, and 25% water by mass using a WR-229 waveguide-based fixture over 3.30–4.90 GHz. Descriptors were extracted from the raw and empty-reference-normalized S_11_ and S_21_ responses, including extrema, slopes, area-based indicators, band-averaged values, selected-frequency responses, and phase statistics. Ridge regression, partial least squares regression, support vector regression, Gaussian process regression, random forest, and gradient boosting regression were evaluated. The selected PLS model achieved RMSE = 3.56%, MAE = 2.92%, and R^2^ = 0.826 under leave-one-mixture-level-out group-wise cross-validation, which was used as the primary indicator of generalization to an unseen mixture level. The substantially lower error obtained under repeated-measurement leave-one-out cross-validation primarily reflects within-level repeatability under controlled conditions and should not be interpreted as evidence of universal calibration performance. The results demonstrate that magnitude- and phase-derived microwave descriptors, particularly transmission-based features, provide an interpretable and repeatable framework for water-content estimation under the specific sand type, sample geometry, water source, and laboratory conditions investigated. Further validation using independent preparation batches, different granular materials, measured water conductivity, temperature variation, and field-like conditions is required before practical deployment.

## 1. Introduction

The measurement of moisture content in granular and porous materials is a fundamental demand in geotechnical engineering, hydrology, agricultural monitoring, construction material evaluation, and electromagnetic material characterization. The water content influences the compaction behavior, the effective dielectric properties, the electromagnetic attenuation, the hydraulic behavior, and the mechanical stability in the case of sand, soil, and related particulate media [1,2,3,4]. Conventional gravimetric methods like oven drying are reliable but destructive, time consuming and inapplicable for rapid and repeated in situ monitoring [5,6,7,8,9]. Due to this, there has been a lot of research on dielectric and microwave sensing methods as alternative non-destructive methods for determining the moisture content of soil or granular materials [1,2,3,10,11,12,13,14,15,16].

The principle of microwave moisture measurement is that there is a high dielectric contrast between dry mineral particles and water. The permittivity and loss of dry sand and mineral soils are typically much smaller than those of liquid water, whereas the permittivity and interaction of water with the microwave field are quite large [3,17,18]. Thus, any increase in water content, even modest, can change the effective dielectric constant, loss factor, propagation delay, and attenuation of a soil–water or sand–water mixture. Previous studies by Topp et al. [1] have demonstrated the feasibility of estimating soil water content using the dielectric properties of transmission lines. Hallikainen et al. [2] and Dobson et al. [3] developed important empirical and mixing-model relations for the microwave dielectric properties of wet soils. These studies provide a basis for understanding the microwave responses of mineral–water mixtures as a function of moisture content.

A number of electromagnetic techniques are available for measuring water content, such as time- and frequency-domain reflectometry, capacitance sensors, resonant sensors, radar-based methods, and broadband microwave methods [5,6,7,8,9,10,11,12,16,19,20,21,22,23]. The most common methods are the time domain and the capacitance. The results from these methods are influenced by probe geometry, contact quality, salinity, bulk density, texture, and calibration conditions [6,7,8,9,10,11,12]. Another approach is through microwave S-parameter measurements, which measure both the reflected and transmitted response of a material or sample holder across a frequency range. The reflection parameter S_11_ is related to impedance mismatch and surface interaction, whereas the transmission parameter S_21_ involves attenuation and propagation of the wave through or across the material. Water changes both the dielectric loss and the effective propagation properties of the media in moisture sensitive media, making S_21_ an even more informative measurement [16,19,20,21,22,23,24].

In recent years, more research has been done that integrates microwave sensing with multivariate regression and machine learning. There has been a report indicating that inexpensive microwave soil moisture sensing using S_11_ spectra, PLS regression [15], and wideband radar features has been employed for soil moisture estimation using machine learning [16]. Many broader reviews have focused on the need for data-driven models for the estimation of soil moisture from remote sensing and microwave observations, with caution expressed regarding the reliability of the models, given the calibration design, independent validation, environmental variability, and interpretation of features [12,13]. This is especially important in small or controlled test sets where measurements are repeated in few moisture conditions to produce very high cross-validation scores when the validation procedure does not adequately reflect independent mixture conditions [25,26,27,28].

Partial least squares regression is particularly attractive for S-parameter analysis because microwave-derived predictors are often strongly correlated. Neighboring frequency points, band averages, selected-frequency amplitudes, and normalized spectral descriptors may carry overlapping information about the same physical interaction. PLS transforms these correlated predictors into latent variables that maximize covariance with the target variable and is widely used in chemometrics and spectral regression [29,30,31]. Support vector regression and Gaussian process regression are also common nonlinear regression tools [28,32], but their performance depends on data size, hyperparameter selection, and validation design. For controlled microwave experiments, physically meaningful feature extraction can reduce dimensionality and improve interpretability compared with directly using the entire spectrum [15,16,19,29].

Despite the extensive literature on dielectric moisture measurement, several research gaps remain in the context of controlled granular materials. First, many studies focus on dielectric mixing models, probe-based sensors, capacitance methods, resonant sensors, or radar-based indicators, whereas fewer examine a compact, physically interpretable S-parameter feature workflow for sand–water mixtures measured with a waveguide-based fixture. Second, the direct use of dense microwave spectra can reduce interpretability and increase model complexity, especially in small datasets with highly collinear spectral variables. Third, the relative contributions of reflection- and transmission-based descriptors are not always explicitly identified, even though these two responses represent different electromagnetic interactions. In the present configuration, S_21_ is expected to be particularly informative because it represents the transmitted microwave response through the sample path and is directly affected by water-induced changes in bulk attenuation, effective permittivity, dielectric loss, and propagation behavior. By contrast, S_11_ is more strongly influenced by impedance mismatch, surface interactions, and fixture-related reflections.

Recent studies also show that moisture estimation is increasingly moving toward data-driven and sensor-integrated frameworks. Abdulraheem et al. reviewed recent progress in dielectric-property-based soil water-content measurements and emphasized the importance of dielectric permittivity, empirical models, and mixing models for moisture estimation [12]. Lamichhane et al. reviewed soil-moisture prediction using remote sensing and machine-learning algorithms and highlighted the need for reliable calibration, validation, and interpretable model development [13]. In microwave/radar-based sensing, Uthayakumar et al. demonstrated that wideband radar features combined with machine-learning models can be used for enhanced soil-moisture estimation [16]. Recent studies have also reported the use of ground-penetrating radar with SAR data, low-cost sensors, and salinity-dependent sensor assessment for soil-moisture monitoring. These studies confirm the growing relevance of electromagnetic sensing and machine-learning-assisted moisture estimation while also showing that compact, laboratory-scale, physically interpretable S-parameter feature workflows for controlled sand–water mixtures still require further investigation [33,34].

The present study, therefore, investigates the feasibility of estimating water content in controlled sand–water mixtures using microwave S-parameter measurements and physically derived features. Six mixture levels were studied: dry sand and 5%, 10%, 15%, 20%, and 25% water by mass. Ten repeated measurements were collected for each level, yielding 60 measurements. The measured S_11_ and S_21_ spectra were processed together with empty-reference-normalized responses, and a set of interpretable features was extracted. Ridge regression, PLS regression, SVR, GPR, random forest, and gradient boosting regression were then compared under cross-validation. The study specifically aimed to acquire microwave S_11_ and S_21_ responses of controlled sand–water mixtures over the 3.30–4.90 GHz band using a WR-229-based fixture, extract physically interpretable descriptors from raw and empty-reference-normalized spectra, compare classical and machine-learning regression models for water-content estimation, and identify informative microwave features within an interpretable and repeatable controlled laboratory framework. The results are intentionally interpreted in the context of repeated laboratory measurements from a single sand type and a single preparation batch for each mixture level, rather than as a universal model for all sand types and field conditions.

## 2. Materials and Methods

Six controlled mixture levels were considered in this study. The dry reference level consisted of sand only and was labeled as 0% water content. The sand was used as the laboratory dry-reference material, and no additional oven-drying step was applied before mixture preparation. Consequently, the residual or bound-water content of the initial sand was not independently quantified. The nominal 0% level should therefore be interpreted as the laboratory dry-reference condition rather than as a verified absolute zero-water state. The remaining levels were prepared as 95% sand–5% water, 90% sand–10% water, 85% sand–15% water, 80% sand–20% water, and 75% sand–25% water. All sand–water ratios were defined on a mass basis; for example, the 95% sand–5% water level corresponds to a 95:5 sand-to-water mass ratio. The same water source was used for preparing all sand–water mixtures. However, its electrical conductivity, mineralization level, and detailed ionic composition were not independently measured. Therefore, the present calibration is specific to the water source used under the controlled experimental conditions and should not be interpreted as applicable to waters with different salinity or mineral content. For each mixture level, ten repeated measurements were performed, resulting in a balanced dataset of 60 measurements.

The 0–25% water-content range was selected to represent a controlled laboratory interval from dry sand to a relatively high moisture content while maintaining sample stability and repeatable filling inside the thin holder. Water contents above this range may increase the possibility of free-water accumulation, non-uniform distribution, and reduced repeatability in a thin sample geometry. Therefore, the selected range was considered suitable for evaluating the sensitivity of microwave S-parameter features to water content in controlled sand–water mixtures.

For each water-content level, the required amounts of sand and water were weighed on a mass basis. The sand and water were manually mixed for 5 min to improve the uniformity of water distribution within the granular material. After mixing, the prepared mixture was allowed to rest for 10 min before measurement to reduce short-term redistribution effects and obtain a more stable sample condition. The mixture was then inserted into the PLA holder, and the sample surface was gently levelled to reduce local air gaps, non-uniform filling, and excessive compaction differences. After each measurement, the holder was emptied and refilled for the next repetition. This procedure was used to evaluate the repeatability of the proposed microwave sensing approach under controlled laboratory conditions. The mixture levels used in the microwave measurement dataset are summarized in Table 1.

For each measurement, the microwave reflection and transmission responses were recorded as S_11_ and S_21_ over the 3.30–4.90 GHz frequency range using a LibreVNA vector network analyzer (Wuyi Zeenko Industrial and Trading Co., Ltd., Jinhua, China)-based microwave measurement system.The measurement fixture was based on a WR-229 rectangular waveguide configuration. The WR-229 waveguide has an inner cross-section of approximately 58.17 × 29.08 mm, which defines the transverse aperture of the sample region. This waveguide was selected because its operating frequency range is compatible with the 3.30–4.90 GHz band used in this study and because its aperture provides a practical sample region for granular sand–water mixtures. Compared with smaller waveguides operating at higher frequencies, the WR-229 configuration offers a larger sample aperture and facilitates repeatable filling of granular material. Other waveguides may also be used for microwave moisture sensing at different frequency bands; however, the WR-229-based configuration was suitable for the present laboratory setup, sample geometry, and target frequency range.

The prepared sand–water mixture was placed in a custom PLA holder positioned in the waveguide measurement path. The holder was designed according to the WR-229 aperture, and the nominal sample thickness along the microwave propagation direction was 3 mm. This thin sample thickness was selected to obtain a repeatable filling geometry, avoid excessive attenuation of transmission, and maintain a controlled microwave interaction length through the sand–water mixture. Thus, the microwave signal interacted with a thin and controlled layer of the sand–water mixture during the S-parameter measurement.

An empty-holder/fixture measurement was recorded before the sample measurements and used as the reference response for normalization. The empty-reference-normalized transmission and reflection responses were calculated at a decibel scale by subtracting the empty response from the sample response. This procedure reduced the influence of the waveguide fixture, coaxial-to-waveguide transitions, cables, connectors, and background transmission effects.

All measurements were carried out at room temperature, approximately 25 °C. The frequency sweep was performed using 1001 uniformly spaced frequency points over the 3.30–4.90 GHz band. No signal averaging or post-measurement smoothing was applied before feature extraction. Therefore, the extracted descriptors were calculated directly based on the measured and empty-reference-normalized S-parameter responses. In the present study, the regression features were derived from magnitude- and phase-based S-parameter descriptors rather than by directly using the real and imaginary components of the complex S-parameters. This was an explicit feature-engineering choice intended to retain physical interpretability through attenuation-, reflection-, transmission-, slope-, and phase-related descriptors. It should not be interpreted as implying that alternative complex-domain representations are uninformative. Future studies may compare magnitude/phase-based descriptors with predictors derived directly from the real and imaginary components of S_11_ and S_21_, particularly using independent preparation batches and external validation datasets.

Instead of using the full spectrum directly, a compact set of physically interpretable descriptors was extracted from the raw and normalized S-parameter responses. These features included extrema, corresponding frequencies, dynamic ranges, mean and median values, standard deviations, integral/area descriptors, end-to-end slopes, linear-fit slopes, band-averaged values, selected-frequency values at 3.5, 4.0, and 4.5 GHz, and phase statistics. Band-averaged features were calculated for representative sub-bands of 3.30–3.70 GHz, 3.70–4.10 GHz, and 4.10–4.90 GHz. This feature-extraction strategy reduces the dimensionality of the regression problem and preserves physical interpretability. The main families of physically derived S-parameter features are summarized in Table 2.

**Figure 1:**
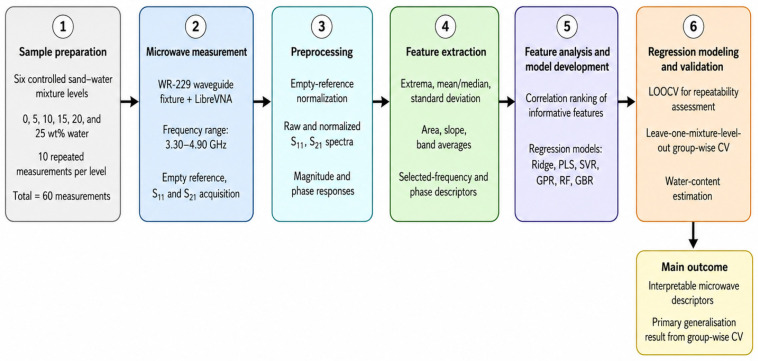
Overall workflow of microwave S-parameter measurement, feature extraction, and regression-based water-content estimation.

Six regression approaches were evaluated to provide both classical and contemporary machine-learning benchmarks: ridge regression, PLS regression, SVR with a Gaussian kernel, GPR, random forest regression, and gradient boosting regression. The target variable was the water content percentage, whereas the predictor variables were the physically derived S-parameter features. Because only 60 repeated laboratory measurements were available and the extracted spectral features were highly collinear, model evaluation was performed using an outer leave-one-out cross-validation (LOOCV) scheme. For tunable models, the hyperparameters were selected inside the training data of each outer fold using inner cross-validation, thereby reducing selection bias and avoiding direct optimization on the left-out test sample. The SVR hyperparameters included the box constraint, kernel scale, and epsilon value; random forest and gradient boosting were tuned over the number of trees and minimum leaf size, with the learning rate additionally tuned for the boosting model. PLS was evaluated over a range of latent-variable numbers, and the final component number was selected according to cross-validation performance. In addition, the robustness of the selected PLS model was examined using 100 repeated stratified 70/30 train/test partitions. To provide a stricter assessment of model generalization, an additional leave-one-mixture-level-out group-wise cross-validation strategy was also implemented. In this validation scheme, all 10 repeated measurements belonging to one water-content level were withheld together as the test set, while the remaining five mixture levels were used for training. This procedure was repeated until each water-content level had been used once as the withheld group. This validation setting is more challenging than standard LOOCV because the model is evaluated on an unseen mixture level rather than on another repeated measurement from a level already represented in the training data. PLS component selection was also re-evaluated under this group-wise validation scheme by testing 1–10 latent variables. A compact PLS-4 model was selected as a parsimonious configuration within the 3–5 component range suggested for reducing model complexity. In the present study, the leave-one-mixture-level-out result was treated as the primary indicator of generalization performance because it evaluates prediction for an unseen mixture level. By contrast, LOOCV was interpreted mainly as an assessment of repeatability among repeated measurements from mixture levels already represented in the training data. The overall workflow of the proposed microwave S-parameter measurement, feature-extraction, and regression-based water-content estimation framework is shown in Figure 1.

The microwave data processing chain was mathematically formulated to make the proposed sensing framework reproducible. Let (f_i_) denote the (i)th measured frequency point, where (i = 1, 2, …, N). Since the S-parameter magnitudes were processed at a decibel scale, the empty-reference-normalized transmission and reflection responses were obtained by subtracting the empty-holder response from each sample measurement.

(1) $$S_{\left\{21,norm\right\}\left(f_{i}\right)}=S_{\left\{21,sample\right\}\left(f_{i}\right)}-S_{\left\{21,empty\right\}\left(f_{i}\right)}$$

(2) $$S_{\left\{11,\mathrm{norm}\right\}\left(f_{i}\right)}={\;S}_{\left\{11,\mathrm{sample}\right\}\left(f_{i}\right)}-{\;S}_{\left\{11,\mathrm{empty}\right\}\left(f_{i}\right)}$$

Several scalar descriptors were then extracted from the raw and normalized spectra. The global mean response and the band-averaged response over a selected frequency interval B were calculated as

(3) $${\bar{\left\{S\right\}}}_{\left\{21\right\}}=\frac{1}{N}\sum_{i=1}^{N}S_{21}\left(f_{i}\right)$$

(4) $${\bar{\left\{S\right\}}}_{\left\{21\right\}}^{B}=\frac{1}{N_{B}}\sum_{f_{i}\in B}S_{21}\left(f_{i}\right)$$

In this study, one of the most informative spectral regions was the 3.70–4.10 GHz band. Area- and slope-based descriptors were also calculated to represent the overall attenuation level and the frequency-dependent trend of the response:

(5) $$A_{S21}=\int_{f_{1}}^{f_{N}}\left|S_{21}\left(f\right)\right|df$$

(6) $$m_{S21}=\frac{S_{21}\left(f_{N}\right)-S_{21}\left(f_{1}\right)}{f_{N}-f_{1}}$$

The extracted features formed the predictor matrix X, whereas the target vector y contained the nominal water-content values of 0%, 5%, 10%, 15%, 20%, and 25%. In PLS regression, the correlated feature matrix is projected onto a smaller set of latent variables. The basic PLS decomposition can be written as

(7) $$X=T\;P^{T}+E$$

(8) y = T q + e

Here, T is the latent score matrix, P is the loading matrix, q is the regression loading vector, and E and e are residual terms. The final water-content prediction can be expressed in regression form as

(9) $$\hat{y}=\beta_{0}+\sum_{j=1}^{p}\beta_{j}x_{j}$$

The model performance was evaluated using RMSE, MAE, and R^2^:

(10) $$RMSE\;=\;\sqrt{\left(\frac{1}{n}\right)\sum_{i=1}^{n}{\left(y_{i}-{\hat{y}}_{i}\right)}^{2}}$$

(11) $$\mathrm{MAE}\;=\left(\frac{1}{n}\right)\sum_{i=1}^{n}\left|y_{i}-{\hat{y}}_{i}\right|$$

(12) $$R^{2}=1-\frac{\sum_{i=1}^{n}{\left(y_{i}-{\hat{y}}_{i}\right)}^{2}}{\sum_{i=1}^{n}{\left(y_{i}-{\bar{y}}_{i}\right)}^{2}}$$

## 3. Results

**Figure 2:**
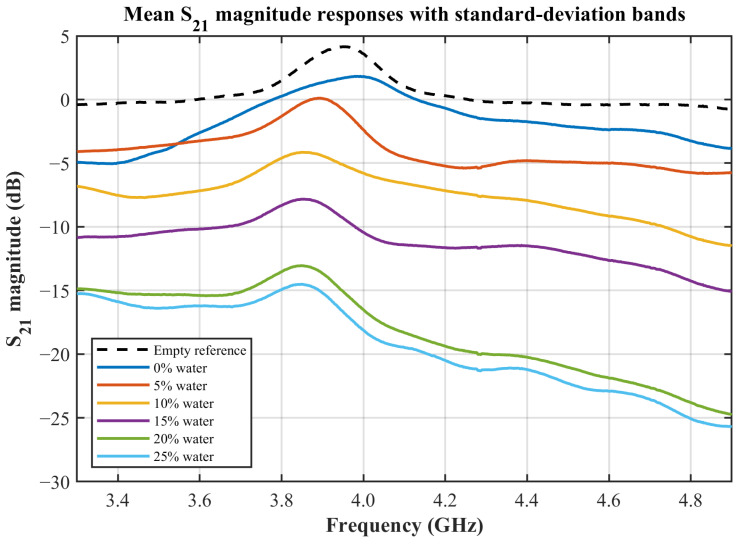
Mean S_21_ magnitude responses of the empty reference and the six sand–water mixture levels, shown with standard-deviation bands based on 10 repeated measurements per group.

**Figure 3:**
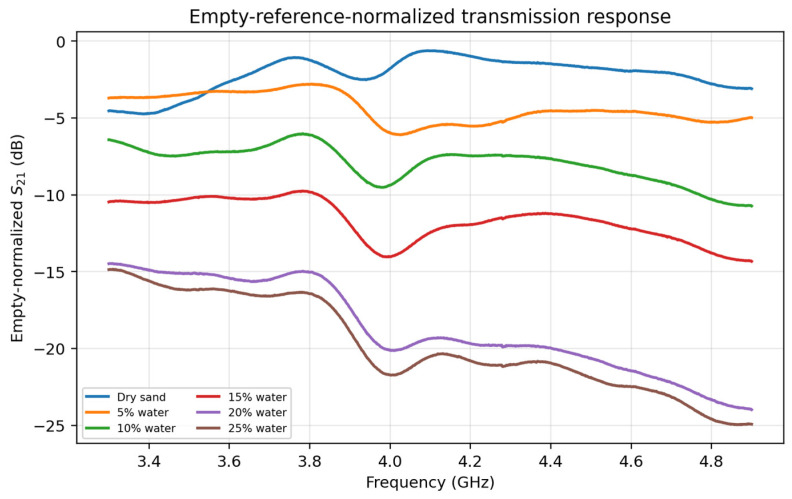
Empty-reference-normalized mean S_21_ responses of the six sand–water mixture levels.

**Figure 4:**
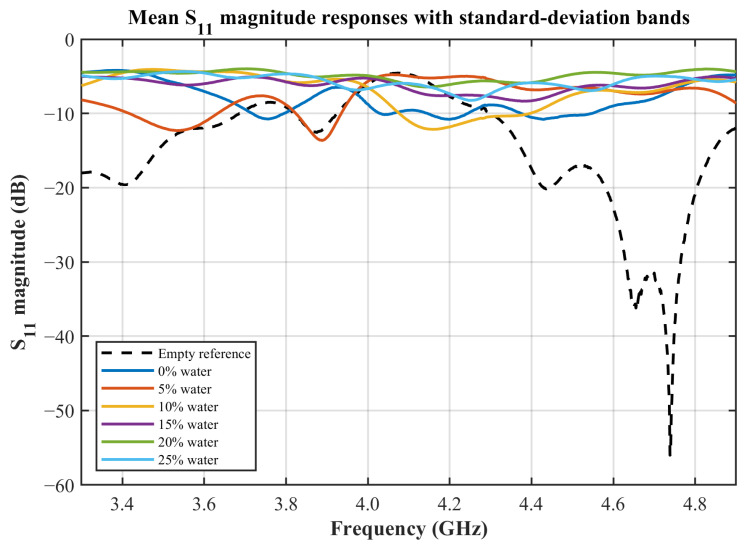
Mean S_11_ magnitude responses of the empty reference and the six sand–water mixture levels, shown with standard-deviation bands based on 10 repeated measurements per group.

The average S_21_ curves showed a clear dependence on water content. As the water fraction increased, the transmission magnitude and its empty-reference-normalized form changed systematically over the measured frequency range. This indicates that microwave propagation through the measurement path was sensitive to the effective dielectric and loss properties of the sand–water mixture. The S_11_ curves also varied with mixture composition; however, the feature-correlation analysis showed that the strongest water-content-related descriptors were dominated by S_21_ based features. This is physically reasonable because S_21_ represents the transmitted response through the sample and is directly affected by water-induced changes in bulk attenuation, effective permittivity, dielectric loss, and propagation behavior, whereas S_11_ is more sensitive to impedance mismatch and fixture-related reflections. Figure 2, Figure 3 and Figure 4 show the mean S_21_ magnitude responses of the empty reference and the six sand–water mixture levels, the corresponding empty-reference-normalized mean S_21_ responses, and the mean S_11_ magnitude responses, respectively.

The extended model comparison results are summarized in Table 3. After inner hyperparameter selection within each outer LOOCV fold, PLS regression produced the lowest numerical prediction error, with RMSE = 0.03%, MAE = 0.02%, and R^2^ = 0.99999. GPR achieved a very similar performance, with RMSE = 0.03%, whereas ridge regression also remained highly accurate despite its simpler linear structure. The nested-CV optimized SVR, random forest, and gradient boosting models produced larger RMSE values than PLS and GPR. Because the dataset consisted of repeated measurements clustered within six controlled water-content levels, the model comparison was interpreted cautiously. Therefore, the results indicate that PLS provided the lowest numerical error in this controlled dataset, but they should not be interpreted as a statistically universal superiority claim over all competing models. The observed behaviour suggests that the relationship between the physically derived microwave features and water content was represented effectively by a compact latent-variable regression model, while the more complex nonlinear and ensemble models did not reduce the prediction error for this small and highly collinear spectral dataset. These LOOCV values should be interpreted as indicators of within-level measurement repeatability rather than as the principal evidence of model generalization. The leave-one-mixture-level-out results reported below provide the more conservative and primary estimate of predictive performance for an unseen mixture level.

To address the effect of repeated measurements within the same mixture level, an additional leave-one-mixture-level-out group-wise cross-validation analysis was performed. In this stricter validation strategy, all 10 repeated measurements from one water-content level were withheld together as the test set, while the remaining five mixture levels were used for training. This setting is more demanding than standard LOOCV because the model is tested on an unseen mixture level rather than on another repetition from a level already represented in the training data. Using the selected parsimonious PLS-4 model, the group-wise cross-validation produced RMSE = 3.56%, MAE = 2.92%, and R^2^ = 0.826. These values are more conservative than the repeated-measurement LOOCV results, confirming that the very high LOOCV R^2^ values mainly reflect within-level measurement repeatability. Accordingly, the group-wise PLS-4 results—RMSE = 3.56%, MAE = 2.92%, and R^2^ = 0.826—are used as the primary performance indicators in the interpretation of model generalization. The PLS component-selection results obtained under leave-one-mixture-level-out group-wise cross-validation are presented in Table 4.

Although PLS-2 yielded the lowest numerical group-wise RMSE, PLS-4 was retained as the predefined compact configuration within the 3–5 component range evaluated during peer review. The modest difference between the PLS-2 and PLS-4 results does not alter the overall interpretation of the controlled feasibility analysis. Both configurations are substantially more parsimonious than the previously evaluated 15-component model.

To improve model transparency and reproducibility, the regression coefficients of the selected PLS-4 model were calculated using the complete feature matrix. The ten predictors with the largest absolute standardized coefficients are reported in Table 5, together with bootstrap standard errors and 95% confidence intervals.

**Figure 5:**
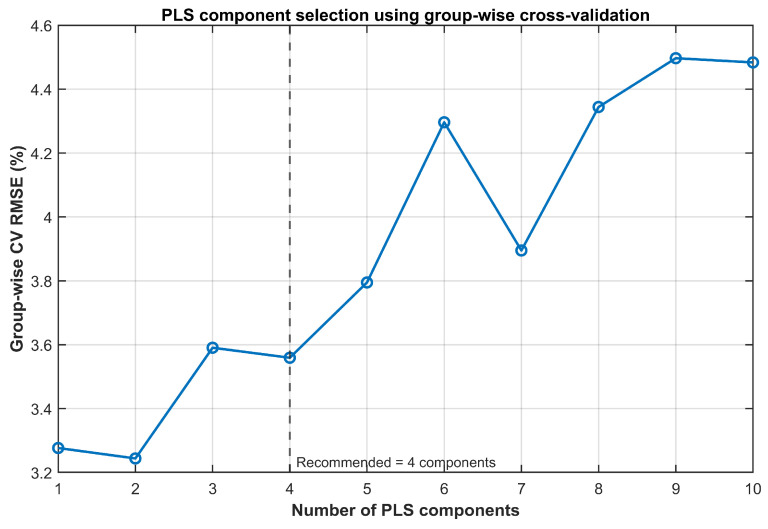
PLS component evaluation using leave-one-mixture-level-out group-wise cross-validation. The dashed vertical line indicates the retained PLS-4 configuration used for the primary model comparison.

**Figure 6:**
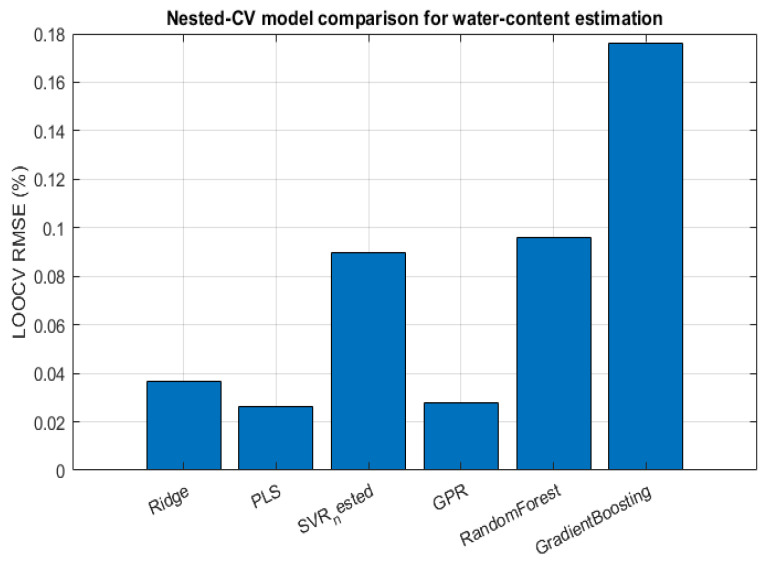
Outer-LOOCV RMSE comparison of the evaluated regression models after inner hyperparameter selection, where applicable.

**Figure 7:**
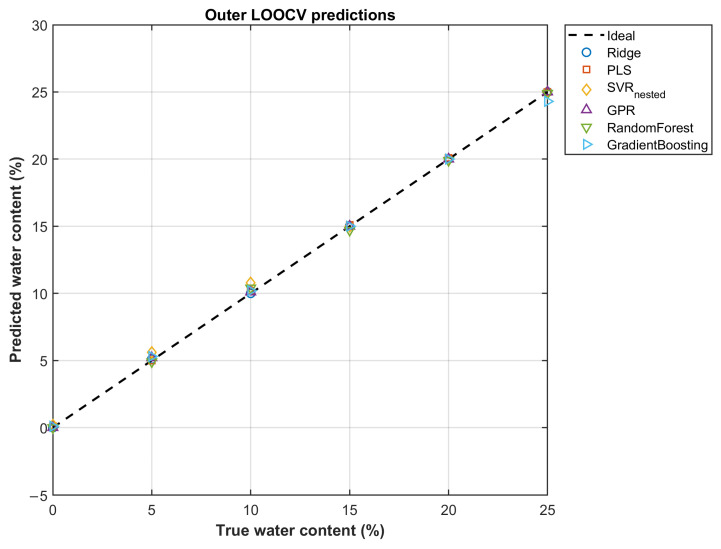
Measured versus predicted water content under outer LOOCV for all evaluated regression models.

Only the 10 predictors with the largest absolute standardized coefficients are shown in the main text for compactness. Only the ten predictors with the largest absolute standardized coefficients are shown in the main text for compactness. These predictors were used to support model interpretability and to identify the most influential microwave descriptors in the selected PLS-4 model. The PLS component-selection results obtained using leave-one-mixture-level-out group-wise cross-validation and the outer-LOOCV RMSE comparison of the evaluated regression models after inner hyperparameter selection are shown in Figure 5 and Figure 6, respectively. The measured versus predicted water-content results obtained under outer LOOCV for all evaluated regression models are shown in Figure 7.

Because PLS provided the lowest numerical LOOCV error while retaining strong interpretability, its robustness was examined using both repeated stratified train/test validation and the stricter leave-one-mixture-level-out group-wise validation. The repeated stratified train/test analysis evaluates stability when all water-content levels are represented in both training and testing subsets, whereas the group-wise validation evaluates prediction on an unseen water-content level. Therefore, the group-wise validation provides a more conservative estimate of model generalization. The repeated stratified train/test validation results of the PLS model are summarized in Table 6.

The repeated train/test results confirmed that the PLS model remained stable under random stratified partitioning. The mean test RMSE was 0.0068%, the mean test MAE was 0.0050%, and the mean test R^2^ was 0.9999988. These values are slightly higher than the LOOCV error, as expected for a more restrictive validation strategy, but they still indicate that the extracted microwave S-parameter features provide highly repeatable information about the controlled water-content levels.

The group-level summary in Table 7 confirms that the PLS predictions were very close to the nominal water-content values for all six mixture levels. The mean predicted values were close to the target values for every group, and the absolute and root-mean-square errors remained very small. This behavior demonstrates high repeatability of the measurement-feature-model chain under controlled laboratory conditions.

**Figure 8:**
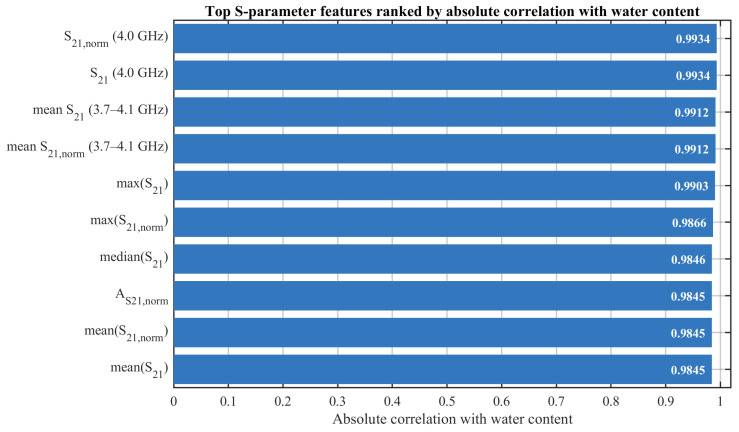
Ten most correlated physically derived features with water content.

The correlation analysis summarized in Table 8 showed that the most informative variables were mainly transmission-based. The strongest feature was the empty-reference-normalized S_21_ value at 4.0 GHz, followed by the raw S_21_ value at 4.0 GHz. The mean S_21_ response in the 3.70–4.10 GHz band and its normalized equivalent also showed very high correlations with water content. This result is physically consistent with microwave propagation in moisture-sensitive media: increasing water content modifies dielectric loss and effective permittivity, which in turn alters transmission attenuation and band-averaged S_21_ behavior. The dominance of S_21_-based descriptors also suggests that transmission measurements are more directly sensitive to water-induced bulk changes than reflection-only descriptors in the present measurement configuration. The top 10 S-parameter features ranked by absolute correlation with water content are shown in Figure 8.

To provide an interpretable single-feature calibration check, the most correlated feature, $S_{21,norm}$ at 4.0 GHz, can also be represented using a simple linear calibration model:

(13) $$W\;=\;a\;*\;S_{S21,\mathrm{norm}\left(4.0\;\mathrm{GHz}\right)\;}+\;b$$

where W is the estimated water content and a and b are calibration coefficients. Using the group-mean values, the fitted relation was W = −1.1771× S_21,norm(4.0 GHz)_ − 1.8095 with R^2^ = 0.9868. This one-feature expression is not intended to replace the multivariate PLS model, but it provides a physically interpretable calibration check by showing how the transmission response at a single informative frequency varies with water content.

**Figure 9:**
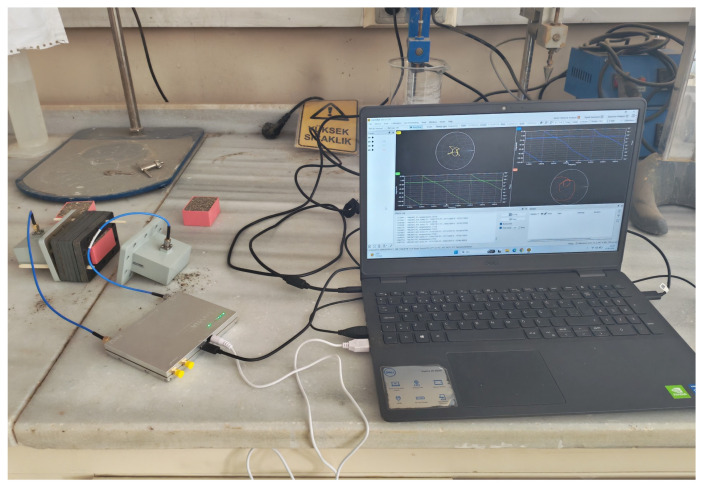
Photograph of the experimental microwave measurement arrangement, including the laptop-based acquisition interface, LibreVNA device, coaxial connections, coaxial-to-waveguide transitions, WR-229-based sample fixture, and the custom holder containing the sand–water mixture. The holder was designed according to the WR-229 aperture, and the nominal sample thickness along the microwave propagation direction was 3 mm.

For completeness, the practical laboratory arrangement used in the measurements is shown in Figure 9. The setup included a laptop-based acquisition interface, a LibreVNA device, coaxial connections, coaxial-to-waveguide transitions, and a WR-229-based custom sample measurement fixture. The sand–water mixture was placed in a thin sample holder located in the waveguide measurement region. The holder matched the WR-229 aperture, while the nominal sample thickness along the propagation direction was 3 mm. Although the main prediction model relied on multiple physically derived features, an additional repeatability-oriented analysis was carried out using the single most informative descriptor, the normalized *S*_21_ value at 4.0 GHz.

Group-wise statistics of the normalized S_21_ response at 4.0 GHz are summarized in Table 9. The standard deviations were very small for all six groups, and the coefficients of variation remained below 0.4%, indicating high measurement repeatability within each nominal water-content level.

**Figure 10:**
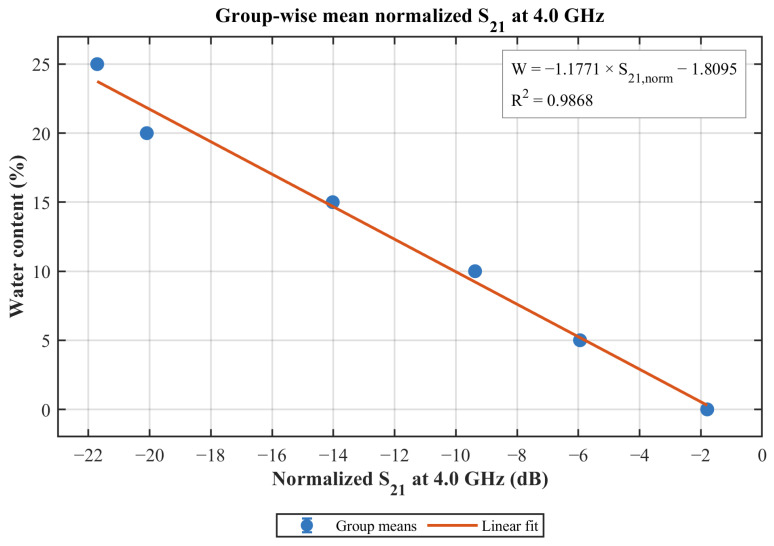
Group-wise mean normalized S_21_ at 4.0 GHz with standard-deviation error bars and the corresponding linear calibration line.

The mean normalized S_21_ values at 4.0 GHz followed an almost monotonic trend with increasing water content, as shown in Figure 10. A simple linear fit based on the group means yielded W = −1.1771 × S_21,norm(4.0 GHz)_ − 1.8095 with R^2^ = 0.9868. This single-feature relation is less general than the multi-feature PLS model, but it provides a compact and physically interpretable calibration check.

**Figure 11:**
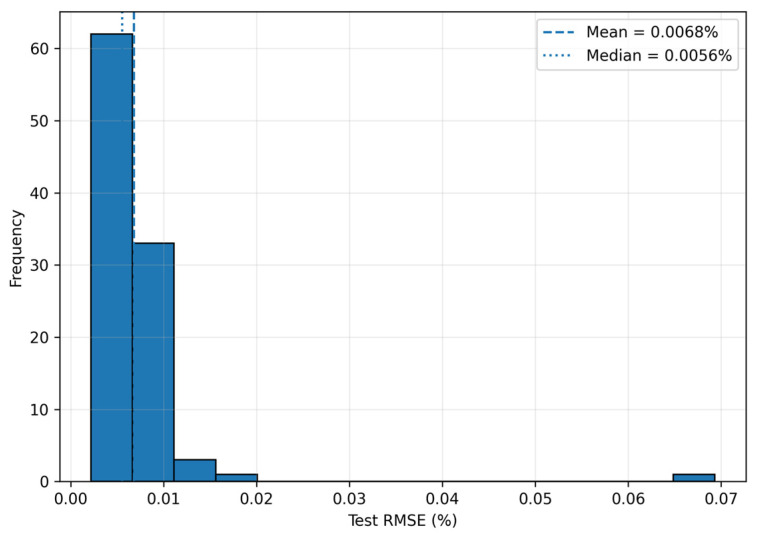
Distribution of test RMSE values from the 100 repeated stratified train/test partitions of the PLS model.

The distribution of test RMSE values obtained from the 100 repeated stratified train/test partitions is shown in Figure 11. Most repetitions clustered in a very narrow low-error range, with only a limited spread, further supporting the robustness of the PLS-based regression framework.

## 4. Discussion

The experimental results show that microwave S-parameter features are sensitive to differences among the controlled sand–water mixture levels. This behavior is consistent with the general principle of microwave moisture sensing, in which the addition of water modifies the effective permittivity, dielectric loss, and propagation characteristics of mineral–water mixtures. Classical dielectric studies on wet soils and transmission-line-based moisture measurements have shown that the electromagnetic response of granular and porous media is strongly affected by water content. The present study supports this physical interpretation using a waveguide-based S-parameter measurement framework and shows that physically derived spectral descriptors can be used for repeatable water-content estimation under controlled laboratory conditions.

The dominant role of S_21_-based descriptors is an important outcome of the study. In the present fixture configuration, S_21_ represents the transmitted microwave response through the sample path and is therefore directly related to water-induced changes in bulk attenuation, effective permittivity, dielectric loss, and propagation behavior. This explains why S_21_ at 4.0 GHz, the mean S_21_ response over the 3.70–4.10 GHz band, and their empty-reference-normalized forms showed the strongest correlations with water content. By contrast, S_11_ is more sensitive to impedance mismatch, surface interaction, and fixture-related reflections. Therefore, although S_11_ also changed with mixture composition, the main predictive information was carried by transmission-based features. This finding agrees with the physical expectation that bulk moisture variation is more directly reflected in the transmitted response than in reflection-only descriptors for the present sample geometry.

The low numerical error obtained by PLS regression is physically and statistically reasonable for the present dataset. The extracted S-parameter features are highly correlated because they are derived from the same underlying spectra and adjacent frequency regions. PLS is specifically designed for such collinear predictor spaces because it projects the original variables onto latent directions that maximize covariance with the response. The additional inclusion of nested-CV optimized SVR, GPR, random forest, and gradient boosting regression strengthens the model comparison. However, the comparison should be interpreted cautiously. GPR achieved a performance very close to PLS, and the dataset consisted of repeated measurements clustered within six controlled water-content levels. Therefore, the results indicate that PLS provided the lowest numerical error in the present controlled dataset, rather than proving a statistically universal superiority over all competing models.

The extremely high LOOCV accuracy should be interpreted carefully. In LOOCV, a single repeated measurement is left out while the remaining measurements from the same water-content level remain in the training set; therefore, the problem is easier than prediction on completely independent mixtures. The use of inner hyperparameter selection for tunable models reduces direct test-fold leakage, but it does not remove the fact that repeated measurements from the same controlled mixture levels are highly clustered. The weaker performance of the nested-CV optimized SVR compared with PLS and GPR may be related to this structure: even after tuning the box constraint, kernel scale, and epsilon values, the Gaussian-kernel SVR can be sensitive to tightly clustered repeated measurements and highly correlated predictors. Therefore, the results should be interpreted as a controlled laboratory feasibility demonstration rather than a fully generalized field calibration.

The additional group-wise cross-validation analysis provides a more realistic interpretation of the predictive performance. In the original LOOCV setting, only one repeated measurement was left out at a time, while other measurements from the same water-content level remained in the training set. Therefore, the very high LOOCV R^2^ values should be interpreted mainly as evidence of measurement repeatability and within-level consistency. In contrast, the group-wise validation withheld all repeated measurements from one mixture level together and tested the model on an unseen water-content level. As expected, the prediction error increased and the R^2^ value decreased to 0.826 for the selected PLS-4 model. This confirms that the proposed framework retains meaningful predictive information under controlled conditions but should not be overinterpreted as a fully generalized calibration model. The stricter validation result provides a more conservative and transparent assessment of model generalization.

Compared with previous microwave and dielectric moisture-sensing studies, the very low LOOCV error observed here reflects the repeated-measurement structure of the controlled dataset and should primarily be interpreted as evidence of within-level repeatability. The leave-one-mixture-level-out group-wise validation provides the more appropriate estimate of generalization to an unseen mixture level. Under this stricter strategy, the selected PLS-4 model achieved an RMSE of 3.56%, an MAE of 2.92%, and an R^2^ of 0.826. These results show that the feature-based microwave framework retains meaningful predictive information under controlled conditions, but they do not establish a universal or field-ready calibration model.

The measurement-system response should also be considered when interpreting the raw S-parameter curves. In Figure 2, small positive S_21_ values observed for the empty reference and dry sample should not be interpreted as material gain. They are attributed to the complete fixture response, reference-plane placement, coaxial-to-waveguide transitions, coupling conditions, and residual mismatch in the measurement path. For this reason, an empty-holder/fixture measurement was recorded and used for reference normalization. Similarly, the empty-reference S_11_ response in Figure 4 indicates that some residual mismatch remained at certain frequencies. This may affect reflection-based interpretation; however, the strongest predictive descriptors identified in this study were dominated by S_21_-based features. Therefore, the main conclusions are primarily supported by transmission-related attenuation and propagation information rather than by reflection-only behavior.

Before broader application, further validation using independently prepared batches, different sand types and granulometries, variable compaction densities, wider moisture intervals, temperature-dependent measurements, salinity variation, and field-like environmental conditions is required. External test sets and group-wise validation strategies that withhold complete mixture levels or preparation batches would provide a more rigorous assessment of interpolation and extrapolation capability. The present results provide an initial controlled feasibility demonstration and identify S_21_-based descriptors that may guide future microwave sensing investigations.

The present study should be interpreted as a controlled laboratory feasibility investigation rather than as a field-ready or universally generalizable calibration model. The dataset was obtained from one sand source, one water source, six nominal water-content levels, a fixed 3 mm sample geometry, and repeated measurements prepared under controlled laboratory conditions. The method was not evaluated using independently prepared batches, natural soils, different sand granulometries, variable compaction states, or field environmental conditions.

Several material-characterization limitations should also be considered. First, the electrical conductivity, mineralization level, and detailed ionic composition of the water source were not independently measured. Second, the initial sand was not oven-dried, and its residual or bound-water content was not quantified; therefore, the nominal 0% level represents the laboratory dry-reference condition rather than a verified absolute zero-water state. Third, all measurements were conducted at approximately 25 °C, and temperature-dependent dielectric variation was not systematically evaluated. These limitations restrict the present calibration to the specific material source, preparation protocol, sample geometry, and laboratory conditions used in this study. Future work should include independent preparation batches, measured water conductivity and salinity, verified baseline moisture, different granular materials, variable temperature and compaction conditions, and external field-like validation datasets.

## 5. Conclusions

This study demonstrated the feasibility of estimating nominal water content in controlled sand–water mixtures using WR-229 waveguide measurements, empty-reference normalization, physically interpretable S-parameter descriptors, and partial least squares regression. Transmission-based features, particularly selected-frequency and band-averaged S_21_ descriptors, were the most informative predictors under the present measurement configuration.

The leave-one-mixture-level-out group-wise validation produced an RMSE of 3.56%, an MAE of 2.92%, and an R^2^ of 0.826 for the selected PLS-4 model and is considered the primary indicator of generalization performance in this study. The substantially lower error obtained under repeated-measurement LOOCV primarily reflects repeatability within mixture levels already represented in the training data.

The methodological contribution is therefore an interpretable and repeatable feature-engineering and regression framework demonstrated under controlled laboratory conditions, rather than a universal calibration or field-ready sensing solution. The results are limited to one sand source, one water source, six nominal water-content levels, a fixed sample geometry, and measurements performed at approximately 25 °C. Water conductivity and mineralization were not independently measured, residual or bound water in the initial sand was not quantified, and temperature dependence was not systematically investigated.

Future work should include independently prepared batches, different sand types and granulometries, measured water conductivity and salinity, verified baseline moisture, variable temperature and compaction conditions, and external field-like validation datasets. Such studies are required before the framework can be considered for practical deployment or broader calibration.

## Figures and Tables

**Table 1:** Mixture levels used in the microwave measurement dataset.

Group	Nominal Water Content (%)	Number of Repeated Measurements	Total Records per Group
Dry sand	0	10	10
95% sand + 5% water	5	10	10
90% sand + 10% water	10	10	10
85% sand + 15% water	15	10	10
80% sand + 20% water	20	10	10
75% sand + 25% water	25	10	10

**Table 2:** Main families of physically derived S-parameter features.

Feature Family	Examples	Physical Meaning
Extrema	Minimum S_21_, maximum S_21_, frequency of minimum S_21_, frequency of maximum S_21_	Peak attenuation or transmission behavior
Central tendency	Mean S_21_, median S_21_, mean normalized S_21_	Overall level of reflected/transmitted response
Dispersion	Standard deviation of S_21_, range of S_21_, range of normalized S_21_	Repeatability and spectral variability
Area/integral	Area of absolute S_21_, area of absolute normalized S_21_	Cumulative spectral attenuation or response level
Slope	End-to-end S_21_ slope, linear-fit S_21_ slope	Frequency-dependent trend
Band average	Mean S_21_ over 3.70–4.10 GHz, mean normalized S_21_ over 3.70–4.10 GHz	Average response in moisture-sensitive frequency regions
Selected-frequency response	S_21_ at 4.0 GHz, normalized S_21_ at 4.0 GHz	Response at representative frequencies
Phase descriptors	Mean phase of S_21_, standard deviation of phase of S_21_, phase slope of S_21_	Propagation-related information

**Table 3:** Outer-LOOCV prediction performance of the evaluated regression models after inner hyperparameter selection, where applicable.

Model	RMSE (%)	MAE (%)	R^2^
Ridge regression	0.04	0.03	0.99998
PLS regression	0.03	0.02	0.99999
SVR (nested-CV tuned)	0.09	0.04	0.99989
GPR	0.03	0.02	0.99999
Random forest	0.10	0.05	0.99987
Gradient boosting	0.18	0.03	0.99958

**Table 4:** PLS component selection under leave-one-mixture-level-out group-wise cross-validation.

PLS Components	Group-Wise RMSE (%)	Group-Wise MAE (%)	Group-Wise R^2^
1	3.28	2.56	0.853
2	3.24	2.37	0.856
3	3.59	3.04	0.823
4	3.56	2.92	0.826
5	3.79	3.33	0.803
6	4.30	3.36	0.747
7	3.90	3.08	0.792
8	4.34	4.02	0.741
9	4.50	4.28	0.723
10	4.48	4.11	0.724

**Table 5:** Main predictors with the largest absolute standardized coefficients in the selected PLS-4 model.

Feature	Standardized Coefficient	Bootstrap SE	95% CI Lower	95% CI Upper
slopeS_11_	−0.480	0.019	−0.500	−0.426
slopeS_11,norm_	−0.480	0.019	−0.500	−0.426
stdS_21Phase_	0.417	0.019	0.373	0.450
mean_S21Phase_	−0.406	0.037	−0.474	−0.328
diffS_21,band2,band1_	−0.395	0.010	−0.402	−0.363
maxS_11_	−0.338	0.039	−0.414	−0.262
f_Max,S21_	−0.338	0.017	−0.365	−0.300
f_Min,S21_	0.304	0.015	0.266	0.324
S_21_ at4.0 GHz	−0.297	0.009	−0.306	−0.271
S_21,norm_ at4.0 GHz	−0.297	0.009	−0.306	−0.271

**Table 6:** Repeated stratified train/test validation performance of the PLS model over 100 random partitions.

Metric	Mean	Standard Deviation	Minimum	Maximum
Test RMSE (%)	0.0068	0.0067	0.0022	0.0693
Test MAE (%)	0.0050	0.0032	0.0020	0.0330
Test R^2^	0.9999988	0.0000065	0.9999341	0.9999999
Validation setting	100 repetitions	70% train/30% test	stratified by water level	PLS model

**Table 7:** Group-level summary of PLS predictions and errors.

Water Content (%)	*n*	Mean Predicted (%)	Std Predicted (%)	MAE (%)	RMSE (%)
0	10	0.00009	0.00229	0.00178	0.00217
5	10	4.99984	0.00427	0.00301	0.00405
10	10	9.99989	0.00274	0.00207	0.00260
15	10	15.00006	0.00233	0.00158	0.00221
20	10	19.99992	0.00299	0.00232	0.00284
25	10	24.99986	0.00325	0.00267	0.00309

**Table 8:** Top S-parameter features ranked by absolute correlation with water content.

Feature	|Correlation with Water Content|
S_21,norm__(4.0 GHz)	0.9934
S_21__(4.0 GHz)	0.9934
mean S_21_ (3.7–4.1 GHz)	0.9912
mean S_21,norm_ (3.7–4.1 GHz)	0.9912
Max(S_21_)	0.9903
Max(S_21,norm_)	0.9866
Median (S_21_)	0.9846
A_S21,norm_	0.9845
meanS_21,norm_	0.9845
meanS_21_	0.9845
A_S21_	0.9819
mean S_21_ (4.1–4.9 GHz)	0.9817
mean S_21,norm_ (4.1–4.9 GHz)	0.9817
Median (S_21,norm_)	0.9798
S_21_ (4.5GHz)	0.9795

**Table 9:** Group-wise repeatability statistics for the normalized S_21_ response at 4.0 GHz.

Water Content (%)	Mean Normalized S_21_ (4.0 GHz (dB))	Std (dB)	CV (%)
0	−1.7918	0.0063	0.352
5	−5.9454	0.0072	0.121
10	−9.3722	0.0088	0.094
15	−14.0209	0.0067	0.048
20	−20.0935	0.0170	0.085
25	−21.7134	0.0157	0.073

## Data Availability

The data presented in this study are available on request from the corresponding author.
